# Acute and Chronic Effects of Isometric Handgrip Exercise on Cardiovascular Variables in Hypertensive Patients: A Systematic Review

**DOI:** 10.3390/sports5030055

**Published:** 2017-08-01

**Authors:** Breno Q. Farah, Antônio H. Germano-Soares, Sergio Luiz C. Rodrigues, Camila X. Santos, Sávio S. Barbosa, Lauro C. Vianna, Véronique A. Cornelissen, Raphael M. Ritti-Dias

**Affiliations:** 1 Graduate Program in Physical Education. University of Pernambuco, Recife, Pernambuco 50100-010, Brazil; brenofarah@hotmail.com (B.Q.F.); henrique_soares1991@hotmail.com (A.H.G.-S.); scahu72@hotmail.com (S.L.C.R.); camiximenes@hotmail.com (C.X.S.); 2 Group Research in Health and Sport—ASCES College, Caruaru, Pernambuco 55016-901, Brazil; 3 Department of Physical Education and Sport Sciences, Federal University of Pernambuco, Vitoria de Santo Antão, Pernambuco 55608-680, Brazil; savio.barbosa2011.1@gmail.com; 4 Faculty of Physical Education University of Brasília, Brasilia, Distrito Federal 70910-900, Brazil; lcvianna@unb.br; 5 Department of Rehabilitation Sciences, Faculty of Movement and Rehabilitation Sciences, KU Leuven, 3000 Leuven, Belgium; veronique.cornelissen@kuleuven.be; 6 Albert Einstein Hospital, Sao Paulo, São Paulo 06455-010, Brazil

**Keywords:** hypertension, cardiovascular variables, exercise

## Abstract

The aim of this study was to describe, through a systematic review, the acute and chronic effects of isometric handgrip exercise on cardiovascular variables in hypertensive individuals. In this systematic review, we included studies that analyzed whether a single bout or a program with isometric exercises affect cardiovascular variables in hypertensive adults. The electronic database PubMed/Medline was searched for relevant studies published until May 2017. Of the 2927 studies initially identified, 2916 were excluded based on title and abstract and five on the basis of full-text assessment, leaving six studies remaining. In addition, one further study cited in the references of the included articles was included in this review, totaling seven studies included (five studies on the chronic effects of isometric handgrip exercise on cardiovascular parameters). None of the acute studies observed post-exercise hypotension. The majority of the chronic studies found decreases in office blood pressure after isometric handgrip training, with training ranging from 6 to 10 weeks, while heart rate variability parameters were improved in one study and did not change in another. Reduction in oxidative stress was observed; however, this variable was only analyzed in one study. In hypertensives, acute responses to isometric handgrip exercise are very limited due to the small number of studies, therefore more research is required. Furthermore, chronic isometric handgrip training reduces blood pressure; however, there is still a gap in the knowledge on the effects of this modality of exercise on other cardiovascular variables—such as endothelial function, oxidative stress, and cardiac autonomic modulation—which should be addressed in future studies.

## 1. Introduction

Hypertension affects more than 1 billion people [1], accounting for 13% of total deaths worldwide [2]. Interventions to decrease blood pressure have been extensively studied [3,4,5,6,7,8], and among them, lifestyle modifications are a cornerstone in hypertensive subjects [9,10].

Meta-analytical studies [3,4,5,6,7,8] have demonstrated that isometric training decreases office blood pressure, and a recent estimate showed reductions of approximately 7 mmHg for systolic and 5 mmHg for diastolic in hypertensives [11]. Interestingly, these decreases seem to be greater than those observed after aerobic training, the standard mode of exercise recommended for hypertensives [3], although the American Heart Association categorizes isometric exercise as having Level of Evidence C for blood pressure-lowering efficacy in hypertensives, lower than aerobic and dynamic resistance exercise (levels of evidence A and B, respectively) [12].

To date, no meta-analytical study or systematic review has focused on the acute effects of isometric exercise on cardiovascular variables in hypertensive subjects. Since an acute bout of exercise can transiently improve cardiovascular homeostasis, as well as predict chronic effects of training [13,14], it is important to summarize the current literature on this topic.

In addition to office blood pressure, exercise interventions have been shown to improve other cardiovascular variables highly related to cardiovascular risk, including endothelial function, arterial stiffness, and ambulatory blood pressure [15]. However, to date, no study has summarized the effects of isometric handgrip exercise on these cardiovascular variables. This type of forward thinking will help to provide evidence for this time-efficient and cost-effective hypertension therapy. In addition, handgrip exercise is easy to perform and may be accomplished in various locations such as hospitals and in the home, enabling increased adherence to treatment, different from other exercises, which could help health professionals in the treatment of hypertension.

Therefore, the aim of this report was to describe, through a systematic review, the acute and chronic effects of isometric handgrip exercise on blood pressure and other cardiovascular variables in hypertensive individuals.

## 2. Material and Methods

This review analyzed the acute and chronic effects of isometric handgrip exercise on some important cardiovascular parameters in hypertensive adults. Original studies published in indexed journals in the electronic database PubMed/MedLine (National Library of Medicine) were searched. No language restrictions were imposed.

**Figure 1:**
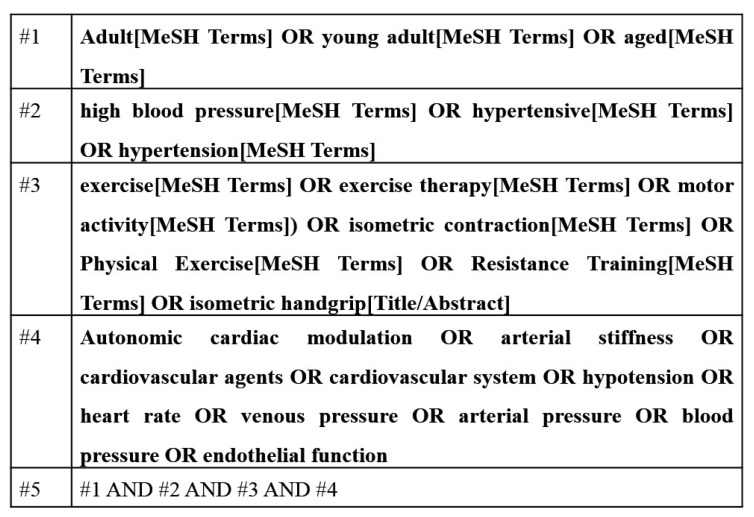
Literature search strategy used for the PubMed database.

The electronic search was performed using advanced tools that allow the combination of descriptors and terms, defined in Medical Subject Headings (MeSH). The search strategy was performed by four blocks of terms such as exercise (exercise OR exercise therapy OR motor activity OR isometric contraction OR physical exercise OR resistance training OR isometric handgrip), outcomes (autonomic cardiac modulation OR arterial stiffness OR cardiovascular agents OR cardiovascular system OR hypotension OR heart rate OR venous pressure OR arterial pressure OR blood pressure OR endothelial function), age (adult OR young adult OR aged), and population (high blood pressure OR hypertension OR hypertensive). Subsequently, the search results were combined (Figure 1). The search involved all available articles published until May 2017. The references of all eligible studies were manually searched for additional studies with potential for inclusion in this review.

Eligibility criteria for inclusion were: (a) isometric handgrip exercise interventions; (b) evaluating at least one cardiovascular parameter; (c) sample including only hypertensive subjects of any age. The sample data extracted from the studies included, but were not limited to, variables related to patient characteristics (sample size, health status, gender, age, and training status), intervention protocol (intensity, weekly frequency, duration, number of sets, and recovery interval), and cardiovascular variables. Study quality was assessed using the Tool for the Assessment of Study Quality and reporting in Exercise (TESTEX) scale [16]. This scale ranges from 0 to 15, and higher scores represent higher methodological quality of the studies. Of note, this scale was originally created for “chronic studies”. Therefore, a modified version of the TESTEX was used for the acute studies, maintaining only the seven pertinent items (eligibility criteria specified, randomization specified, blinding of assessor for at least one key outcome, between-group statistical comparisons reported—2 points, point measures and measures of variability for all reported outcome measures, and exercise volume and energy expenditure). Thus, the score for acute studies ranges from 0 to 7.

Two experienced researchers independently performed the electronic search, article selection, and data extraction and the results were compared. Disagreements were discussed and a third researcher made the final decision.

## 3. Results

**Figure 2:**
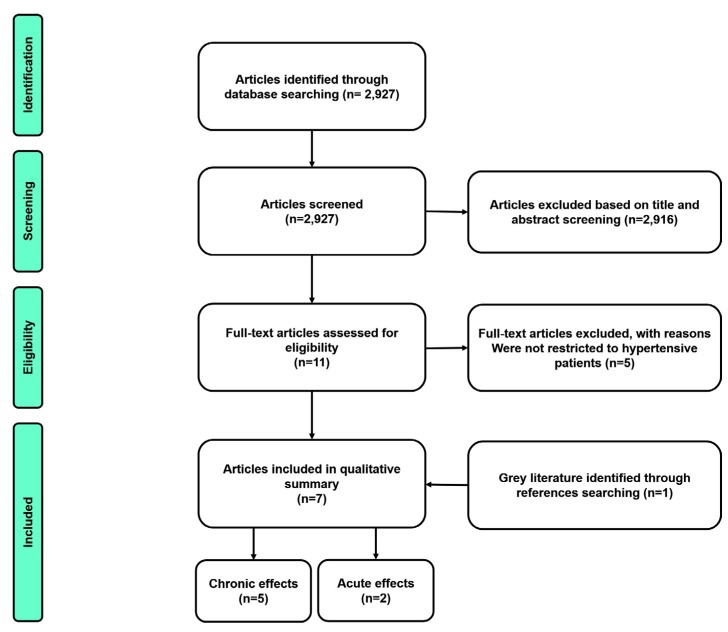
Identification and selection of articles included in the review.

The results of the literature search are presented in Figure 2. Initially, 2927 studies were identified, of which 2916 were excluded on the basis of title and abstract and five on the basis of full-text reading, thus six studies remained eligible for the systematic review. However, one other study cited in the references of the articles was included in this review. Thus, two studies investigated the acute effects of isometric handgrip exercise and five focused on the chronic effects of isometric handgrip exercise on cardiovascular parameters.

Table 1 provides an overview of the study characteristics of the acute trials. Two studies were non-randomized not controlled studies [17,18]. Sample sizes of these studies ranged from 12 to 50 individuals, aged 43 to 64 years and of both sexes. In one study, the hypertensives were unmedicated for at least 20 days [17], the duration of hypertension ranged from 4 months to 9.4 years. In four studies, all patients were treated with hypertensive drugs. Ethnicity was not provided by the studies. One study performed a single exercise session [17], while one included an experimental and a control session [19] (Table 1). One study reached a quality score of 3 (42.9%), while another reached 7 (100%). The cardiovascular variables analyzed in the acute studies were office blood pressure by means of auscultatory and oscillometric techniques (*n* = 2), heart rate (*n* = 2) by ECG, systemic vascular resistance (*n* = 1), cardiac index (*n* = 1) by means of impedance cardiography, and rate pressure product (*n* = 1). The duration of follow-up after the exercise bout was 60 minutes in one study [19] and 24 h in another study [17].

The synthesis of acute studies revealed that systolic blood pressure remained unchanged immediately after exercise [17], while it increased in another study after 60 min [19]. Systemic vascular resistance reduced in hypertensive non-dippers, while no changes in these variables were observed in hypertensive dippers (100%). The two studies that measured heart rate, rate pressure product, and/or the cardiac index did not observe any changes in these variables following a single bout of isometric handgrip exercise (Table 2). 

In the five chronic studies, the number of subjects ranged from 10 to 40 individuals. Four studies were randomized controlled trials [20,21,22,24] and one was a non-randomized, not controlled study [23] (Table 1). The quality of the studies ranged from 6 to 13 (40.0% to 86.7% of the total). The cardiovascular variables analyzed in the chronic studies were office systolic (*n* = 5), diastolic (*n* = 5), and mean blood pressure (*n* = 5); ambulatory blood pressure (*n* = 1); cardiac autonomic modulation indices, such as heart rate variability (*n* = 2); and blood pressure variability (*n* = 1). The vascular variable evaluated was oxidative stress (*n* = 1). Four studies performed four sets with two minutes of contraction, with a one-minute rest interval, at 30% of maximal voluntary contraction, three times per week, while one [23] performed four sets with 45 s of contraction, with a one-minute rest interval, at 50% of maximal voluntary contraction, three times per week. The duration of the training ranged from 6 to 10 weeks (Table 3).

Office systolic blood pressure was significantly reduced in four studies (80.0%) and unchanged in one study. Office systolic blood pressure reductions ranged between 5 to 19 mmHg. Office diastolic blood pressure was significantly lower following isometric handgrip training in one study (6 mmHg) and unchanged in four studies. Mean blood pressure reduced in three studies (60.0%) (3 to 6 mmHg) and was unchanged in two studies. Ambulatory blood pressure did not change after isometric training [22]. One study reported a reduction in blood pressure variability [24], pulse pressure [21], and oxidative stress [23]. Heart rate variability parameters of time, frequency, and non-linear domains did not change in one study [22] (50.0%), while they improved in another (50.0%) (increased low and high frequency [24]). No study reported any adverse effects of isometric handgrip training.

## 4. Discussion

The major findings of this systematic review are three-fold: (a) only two studies investigated the acute effects of isometric handgrip exercise on cardiovascular variables in hypertensives; (b) the only study available suggested that one single bout of isometric handgrip exercises does not decrease office blood pressure; and (c) the majority of the chronic intervention studies reported significant reductions in blood pressure; however, the chronic effects of handgrip training on cardiac autonomic modulation remain unknown.

Our study demonstrates the lack of evidence analyzing the acute effects of a single bout of isometric handgrip exercise on cardiovascular parameters in hypertensive individuals. Two studies were identified [17,19], of which only one included a control session [19]. The study by Olher et al. [19] was the only one to evaluate the acute effects of handgrip exercise on blood pressure and showed no changes up to 60 min after the cessation of exercise. The other study on the acute effects of handgrip exercise [17] showed an increase in the cardiac index in hypertensive patients with dipper and non-dipper patterns; however, systemic vascular resistance increased only in non-dipper hypertensives. Considering that such studies analyzed few cardiovascular variables, understanding of the acute effects of handgrip exercise on the cardiovascular system of hypertensive patients is limited. These aspects reinforce the need for further well-designed studies, aiming to investigate the acute effects of isometric handgrip exercise on cardiovascular variables.

Moreover, there was relative homogeneity regarding the isometric exercise protocols used among the chronic intervention studies. That is, four out of five studies required their participants to complete four sets with two minutes of contraction, with 30% maximum voluntary contraction. Overall, these studies demonstrated reductions in office blood pressure, mainly systolic blood pressure. Only one study [23] used a different protocol (four sets of 45 s at 50% maximum voluntary contraction). Despite this, their results also indicated decreases in blood pressure. Taken together, this suggests that improvements in blood pressure are obtained with different protocols of isometric exercise. The duration of the chronic studies was also similar, ranging from 6 to 10 weeks. Therefore, the effects of different resistance training protocols and the longer-term effects of isometric training remain important gaps in the literature that necessitate further investigation.

Although decreases in blood pressure following handgrip training programs have already been described in previous meta-analytical studies [3,4,5,6,7,8]; this is the first study to summarize the results of the studies that included exclusively hypertensive patients. The majority of the chronic studies presented TESTEX scores over 10, which represent ≥66% of the total scores. Interestingly, the only study analyzing the peripheral effects of isometric training [23] presented a score lower than 6, representing ≤40% of the total. All except one chronic study [22] found decreases in blood pressure after isometric handgrip training. Interestingly, in the only study that did not observe decreases in blood pressure with training [22], the individuals presented the lowest resting BP among all studies, and it is known that decreases in blood pressure after exercise training are greater in those individuals with higher resting blood pressure levels [25]. In the same way, a more recent meta-analysis [8] reported greater mean blood pressure reductions in hypertensives compared to normotensives; however, no differences were found for systolic or diastolic blood pressure.

The studies presented controversial results on the effects of handgrip exercise training on cardiac autonomic modulation. Taylor et al. [24] observed improvement in linear indices of heart rate variability (LF and HF) 10 weeks after isometric handgrip training, which may suggest that a change in autonomic function could be a mediator of blood pressure reduction after this type of exercise [26]. Conversely, Stiller-Moldovan et al. [22] did not observe improvements in cardiac autonomic modulation after isometric training. In the same way, the vascular effects have been poorly studied. The only study [23] that analyzed the effects of handgrip isometric training on biomarkers observed that six weeks of isometric handgrip training improved the glutathione oxidized glutathione ratio, representing a reduction in oxidative stress. However, this study lacked a control group, limiting the strength of the evidence. Based on the inconsistency of these results, the autonomic and vascular mechanisms underlying blood pressure reduction after this modality of exercise in hypertensives are incompletely understood. Moreover, further randomized controlled trials on the effects of this modality of exercise in other potential mechanisms (i.e., cardiac baroreflex sensitivity, flow mediated dilation, shear stress, blood flow, arterial stiffness, ventricular function, oxidative stress, inflammatory markers) are necessary to improve our understanding of the cardiovascular effects of isometric handgrip training in this population.

This study has potential practical applications. The American Heart Association in 2013 classified isometric exercise as Level of Evidence C and Class of Recommendation IIb, due to the absence of studies with hypertensives. However, in this review, we verified that four out of five studies (80.0%) demonstrated reduced office blood pressure after isometric handgrip training, which strengthens the efficacy of this kind of exercise in the treatment of hypertension, in particular, as handgrip isometric exercise may be performed anywhere, is easy to perform, and is of short duration (about 30 min/week). Moreover, no study reported any acute or chronic adverse events of isometric handgrip exercise, demonstrating the safety of this type of exercise. Araujo et al. [18] found a modest increase in heart rate (∆ + 3 ± 4 bpm), systolic blood pressure (Δ + 16 ± 10 mmHg) and diastolic blood pressure (∆ + 7 ± 6 mmHg) in hypertensives during an isometric handgrip exercise protocol. Therefore, health professionals could use isometric handgrip exercise in the treatment of hypertensive patients.

In conclusion, the available literature indicates that acute isometric handgrip exercise does not affect post-exercise blood pressure in hypertensive subjects, although the need for further studies is clear. On the other hand, chronic isometric handgrip training significantly decreases blood pressure. However, given the limited available data on the effects of isometric handgrip training exercise on other cardiovascular variables, such as endothelial function, oxidative stress, and cardiac autonomic modulation, more research is warranted.

## Figures and Tables

**Table 1:** General characteristics of the studies included in the review.

Author (year)	Session/Groups	Age (Years)	Baseline SBP	Baseline DBP	N	Sex	R	Duration of Hypertension	Medicine	Quality Score (%) *
**ACUTE STUDIES**										
Olher et al., (2013) [19]	HS e CS	64 ± 1	121 ± 7	72 ± 6	12	Female	Yes	NI	βb, ACE, Diu, ARB and CCB	7 (100.0)
Porro et al., (1995) [17]	HS	43 ± 3	Ndip—144 ± 3 Dip—134 ± 3	Ndip—96 ± 3 Dip—91 ± 3	50	Both	NA	NI	NI	3 (42.9)
**CHRONIC STDUIES**										
Carlson et al., (2016) [20]	GC GT	CG—54 ± 8 TG—52 ± 8	CG—128 ± 15 TG—136 ± 12	CG—74 ± 9 TG—77 ± 7	CG—20 TG—20	Both	Yes	NI	ACE, Diu, ARB, CCB, αAA and unmedicated	10 (66.7)
Badrov et al., (2013) [21]	GC GT	CG—63 ± 9 TG—65 ± 7	CG—130 ± 17 TG—129 ± 16	CG—73 ± 12 TG—72 ± 9	CG—12 TG—12	Both	Yes	≥4 months	ACE, Diu, CCB, and unmedicated	13 (86.7)
Stiller-Moldovan et al., (2012) [22]	CG TG	CG—63 ± 6 TG—60 ± 8	CG—118 ± 14 TG—114 ± 13	CG—68 ± 4 TG—61 ± 12	CG—9 TG—11	Both	Yes	>4 months	βb, ACE, Diu, ARB and CCB	10 (66.7)
Peters et al., (2006) [23]	TG	52 ± 5	146 ± 11	90 ± 7	10	Both	NA	>6 months	NI	6 (40.0)
Taylor et al., (2003) [24]	CG TG	CG—64 ± 6 TG—69 ± 6	CG—152 ± 8 TG—156 ± 9	CG—87 ± 11 TG—82 ± 9	CG—8 TG—9	Both	Yes	CG—9.2 years TG—9.4 years	βb, ACE, Diu, CCB, and unmedicated	11 (11.3)

R—randomization; NA—not applicable; HS—handgrip session; CS—control session; CG—control group; TG—training group; Ndip—non-dippers; Dip—dippers; NI—not informed; βb—β-blocker; ACE—angiotensin converting enzyme inhibitor; Diu—diuretic; CCB—Calcium channel blocker; ARB—angiotensin receptor blocker; * TESTEX scale score and percentage in relation to the total.

**Table 2:** Characteristics of the physical training program and the main results of the acute studies included in the review.

Author (Year)	Session	Variables Analyzed	Exercise Protocol	Assessment Duration	Main Results
Olher et al., (2013) [19]	HS1, HS2, and CS	SBP, DBP, MBP, HR, and RPP	HS1 = 20 × 10 s; RI NI; 30% MVC. HS2 = 20 × 10 s; RI NI; 50% MVC.	60 min	HS1, HS2 e SC = → SBP, DBP, MBP, HR, and RPP.
Porro et al., (1995) [17]	HS	SVR and CI	1 × 3 min; 30% MVC	24 h	Non-dippers = ↑ SVR and CI Dippers = → SVR; ↑ CI

HS—handgrip session; HS1—HS with 30% of MVC; HS2—HS with 50% of MVC; CS—control session; MVC—maximal voluntary capacity; RI—recovery interval; SBP—systolic blood pressure; DBP—diastolic blood pressure; MBP—mean blood pressure; HR—heart rate; RPP—rate pressure product; SVR—systemic vascular resistance; CI—cardiac index.

**Table 3:** Characteristics of the physical training program and the main results of the chronic studies included in the review.

Author	Variables Analysed	Training Protocol	Frequency	Duration	Main Results
Carlson et al., (2016) [20]	SBP, DBP, MBP, and HR	UN arms:4 × 2 min.; 3 min. RI; 30% MVC.	3 × / weeks	8 weeks	↓ SBP, MBP
Badrov et al., (2013) [21]	SBP, DBP, MBP, and PP	Alternated arms: 4 × 2 min.; 1 min. RI; 30% MVC.	3 × / weeks	10 weeks	↓ SBP, DBP, MBP, and PP (at rest) ↓ SBP (mental and physical stress)
Stiller-Moldovan et al., (2012) [22]	SBP, DBP, MBP (clinic and 24 h), and HRV	Alternated arms: 4 × 2 min.; 1 min. RI; 30% MVC.	3 × / weeks	8 weeks	→SBP, DBP, MBP (clinic and 24 h), and → HRV
Peters et al., (2006) [23]	SBP, DBP, MBP, and oxidative stress	Alternated arms: 4 × 45 s; 1 min. RI; 50% MVC.	3 × /weeks	6 weeks	↓ SBP, and oxidative stress → DBP and MBP
Taylor et al., (2003) [24]	SBP, DBP, MBP, HRV, and BPV	Alternated arms: 4 × 2 min.; 1 min. RI; 30% MVC.	3 × / weeks	10 weeks	↓ SBP and MBP; →DBP; ↑ HRV and BPV

SBP—systolic blood pressure; DBP—diastolic blood pressure; MBP—mean blood pressure; HR—heart rate; HRV—heart rate variability; BPV—BP variability; RI—recovery interval; MVC—maximal voluntary capacity; PP—pulse pressure; UN—uniformed.
